# Mediterranean Diet Adherence and Nutritional Status in Older Adults with Heart Failure with Reduced Ejection Fraction: A Cross-Sectional Study

**DOI:** 10.3390/healthcare14213675

**Published:** 2026-10-30

**Authors:** Yasemin Yetgin, Turgut Karabag, Meral Kucuk Yetgin, Zuhal Aydan Saglam

**Affiliations:** 1 Department of Nutrition and Dietetics, Faculty of Health Sciences, Istanbul Bilgi University, 34060 Istanbul, Türkiye; 2 Department of Cardiology, Istanbul Training and Research Hospital, University of Health Sciences, 34098 Istanbul, Türkiye; turgut.karabag@sbu.edu.tr; 3 Department of Applied Health Science, School of Public Health-Bloomington, Indiana University, Bloomington, IN 47405, USA; mkucukye@iu.edu; 4 Department of Family Medicine, Istanbul Training and Research Hospital, University of Health Sciences, 34098 Istanbul, Türkiye; zuhalaydan.saglam@sbu.edu.tr

**Keywords:** heart failure, mediterranean diet, malnutrition, mini nutritional assessment, MEDAS, older adults, nutrient adequacy, dietary assessment

## Abstract

**Background/Objectives:** Malnutrition is common in older adults with heart failure (HF). We examined the association between Mediterranean diet (MedDiet) adherence and nutritional status in older adults with HF with reduced ejection fraction (HFrEF), their relationship with resting left ventricular ejection fraction (LVEF), and nutrient intake relative to reference values. **Methods:** This cross-sectional study included 125 consecutive outpatients aged ≥ 65 years with HFrEF (LVEF ≤ 40%) in Türkiye, assessed in April–May 2020, during a COVID-19 stay-at-home restriction for this age group. Nutritional status was assessed with the Mini Nutritional Assessment (MNA), MedDiet adherence with the Mediterranean Diet Adherence Screener (MEDAS), and nutrient intake with a single 24 h recall compared with the Estimated Average Requirement (EAR). **Results:** The mean age was 72.9 ± 6.7 years. The MNA classified 15.2% of participants as malnourished and 49.6% as at risk of malnutrition; 30.4%, 43.2%, and 26.4% had low, moderate, and high MedDiet adherence. MNA and MEDAS categories showed a modest association (χ^2^(4) = 11.26, *p* = 0.024; Cramér’s V = 0.21). Each MEDAS point was associated with higher adjusted odds of a better MNA category (odds ratio [OR] 1.40, 95% confidence interval [CI] 1.15–1.72); adjusted ORs were 2.67 (1.12–6.32) for moderate and 4.68 (1.71–12.78) for high versus low adherence. When MNA item D (psychological stress or acute disease) was neutralized, the categorical association was attenuated (*p* = 0.071), whereas the per-point association persisted. LVEF was correlated with neither score. Recall-day intake was below the EAR for vitamin D in 98.4%, folate in 86.4%, and calcium in 84.8% of participants. **Conclusions:** Higher MEDAS scores were associated with better MNA classification in older adults with HFrEF, but neither measure was associated with resting LVEF. Longitudinal studies are needed to establish directionality.

## 1. Introduction

Heart failure (HF) represents the end-stage manifestation of various pathophysiological disorders affecting cardiac function and is characterized by insufficient cardiac output to meet the metabolic demands of body tissues [1]. HF is a common condition among the elderly, with its prevalence increasing with age, ranging from 4.8% to 13.5% in this population [2]. Among elderly patients, heart failure with reduced ejection fraction (HFrEF), defined as a left ventricular ejection fraction (LVEF) ≤40% [3], is particularly concerning due to its association with a poor prognosis, frequent hospitalizations, and diminished quality of life [4].

The prevalence of malnutrition in HF varies between 15% and 90%, depending on the assessment method used. When malnutrition is present, it has been shown to more than double the risk of mortality [5,6,7]. Malnutrition in HF patients is multifactorial, involving reduced appetite, increased energy expenditure, chronic inflammation, and medication-related side effects. It leads to adverse outcomes such as impaired immune function, muscle loss, and higher hospitalization rates [8]. Therefore, nutritional assessment in HF patients not only identifies existing malnutrition but also enables the early detection of metabolic and functional impairments that may influence disease progression.

Diet has long been recognized as a key determinant of cardiovascular health, and in recent years, the focus has shifted from individual nutrients to the synergistic health effects of overall dietary patterns [9]. The Mediterranean diet (MedDiet) is characterized by a high intake of vegetables, fruits, nuts, whole grains, and extra-virgin olive oil; a moderate intake of fish and, sometimes, wine with meals; and a low intake of dairy products, red and processed meats, and free sugars [10]. Its anti-inflammatory and antioxidant properties have been proposed as mechanisms through which it may support cardiovascular function [11].

The strength of the evidence for the MedDiet differs by clinical setting. For the prevention of cardiovascular disease, randomized evidence is available: the Lyon Diet Heart Study reported a substantial reduction in recurrent cardiovascular events with a Mediterranean-type diet after myocardial infarction, with heart failure included among the secondary components of the composite outcomes [12], and the Spanish PREDIMED trial (Prevención con Dieta Mediterránea), which included individuals at high cardiovascular risk, demonstrated that a Mediterranean diet supplemented with extra-virgin olive oil or nuts was associated with an approximately 30% lower risk of major cardiovascular events compared with the control diet [13]; these trials have been critically reviewed elsewhere [14]. For the prevention of incident HF, the evidence is less direct: a meta-analysis of randomized controlled trials including 10,950 participants reported a lower risk of HF with the MedDiet (relative risk 0.30, 95% confidence interval [CI] 0.17–0.56), although this effect was no longer significant after exclusion of a trial with data-integrity concerns [15], and a meta-analysis of six European cohort studies involving 216,385 adults without HF at baseline found that higher adherence was associated with a lower risk of developing HF [16]. In patients with established HF, the evidence is considerably more limited and mainly observational, with associations between MedDiet adherence and symptoms, quality of life, or decompensation that have not been consistently statistically significant [17,18]. The benefits observed in cardiovascular prevention therefore cannot be directly extrapolated to older adults with established HFrEF.

Dietary modifications have a relatively low risk-to-cost ratio and offer an attractive strategy to reduce the incidence and severity of heart failure (HF). Nutritional guidelines related to heart failure typically include modest and nonspecific recommendations, primarily focusing on sodium (Na) and fluid restrictions. However, targeted dietary modifications may have superior effects in the prevention and treatment of HF [17]. From a health-care perspective, nutritional screening and diet-quality assessment are low-cost tools that could be embedded in outpatient HF follow-up.

Although both nutritional status and Mediterranean diet adherence have been investigated in patients with heart failure, these constructs capture different aspects of nutrition: the Mini Nutritional Assessment (MNA) reflects multidimensional nutritional risk, whereas the Mediterranean Diet Adherence Screener (MEDAS) reflects adherence to a specific dietary pattern. Their relationship in older adults with established heart failure with reduced ejection fraction (HFrEF), particularly in a Turkish clinical population, remains insufficiently characterized. In addition, whether nutritional status or Mediterranean diet adherence varies according to the degree of resting left ventricular systolic dysfunction has not been clearly established.

Therefore, this study aimed to characterize nutritional status and Mediterranean diet adherence in older adults with HFrEF, to examine the association between MNA nutritional-status categories and MEDAS adherence categories, and to determine whether either measure was associated with resting left ventricular ejection fraction (LVEF). Dietary nutrient intake was additionally compared with reference values to describe the proportion of participants whose recall-day intake was below the Estimated Average Requirement (EAR). The association between MNA and MEDAS categories was the primary analysis, on which the sample size calculation was based; all other analyses were secondary. We hypothesized that higher Mediterranean diet adherence would be associated with more favorable nutritional status.

## 2. Materials and Methods

### 2.1. Study Design and Setting

This cross-sectional analytical study used both retrospectively obtained clinical data and prospectively collected nutritional and anthropometric data and is reported in accordance with the Strengthening the Reporting of Observational Studies in Epidemiology (STROBE) statement for cross-sectional studies [19]. It was conducted between late April and May 2020 at the Cardiology Clinic of the University of Health Sciences Istanbul Training and Research Hospital and included patients aged ≥ 65 years with heart failure with reduced ejection fraction (HFrEF), defined as a left ventricular ejection fraction (LVEF) ≤40% [3]. This period coincided with the first wave of the COVID-19 pandemic, when a stay-at-home restriction applied to all adults aged ≥ 65 years in Türkiye, with exceptions for essential needs such as medical care; all assessments were performed during in-person clinic visits. The study was approved by the Clinical Research Ethics Committee of the University of Health Sciences Istanbul Training and Research Hospital (decision no. 2240; 27 April 2020) and conducted in accordance with the Declaration of Helsinki.

Inclusion criteria were: (1) age ≥ 65 years, (2) a confirmed diagnosis of HFrEF (LVEF ≤ 40%), and (3) the ability to provide informed consent. Exclusion criteria were: (1) a cancer diagnosis, (2) severe cognitive impairment, (3) an inability to complete assessments, and (4) incomplete data.

Demographic, clinical, and biochemical data were retrospectively obtained from the hospital’s electronic medical records system (PROBEL, İzmir, Türkiye), whereas LVEF was measured by one of the authors (T.K.) using transthoracic echocardiography immediately before the nutritional interview. Echocardiography was performed with the patient in the left lateral decubitus position using a Philips EPIQ 7 system (Philips Healthcare, Andover, MA, USA) with a 2.5–3.5 MHz transducer; M-mode and two-dimensional measurements were obtained from the parasternal long-axis view under electrocardiographic guidance (M-mode recordings at 50 mm/s, Doppler recordings at 100 mm/s). LVEF was calculated using the biplane Simpson method from apical four- and two-chamber views, in accordance with the recommendations of the American Society of Echocardiography and the European Association of Cardiovascular Imaging [20]. All measurements were performed by the same cardiologist to avoid interobserver variability, and the mean of three separate measurements was recorded to reduce intraobserver variability. In line with routine clinical practice, LVEF values were recorded in the medical records rounded to the nearest 5%. Nutritional data, including the 24 h dietary recall, Mini Nutritional Assessment (MNA), and Mediterranean Diet Adherence Screener (MEDAS), were collected prospectively through face-to-face interviews conducted in the hospital by the researcher, a dietitian, immediately after the echocardiographic examination; the interviewer was aware of the participants’ LVEF values. Anthropometric measurements, including body weight, height, waist circumference, and hip circumference, were obtained during the same visit; BMI, waist-to-hip ratio (WHR), and waist-to-height ratio (WHtR) were subsequently calculated.

### 2.2. Participants and Sample Size

An a priori power analysis was conducted using G*Power version 3.1 (Heinrich-Heine-Universität Düsseldorf, Düsseldorf, Germany) to determine the minimum sample size required for the primary analysis. Assuming a medium effect size (w = 0.3), a significance level of α = 0.05, a power of 80% (1 – β = 0.80), and 4 degrees of freedom (based on a 3 × 3 contingency table comparing Mini Nutritional Assessment [MNA] categories with Mediterranean Diet Adherence Screener [MEDAS] levels), the minimum required sample size was calculated as 132 participants. The achieved sample of 125 participants was slightly below this target (power to detect w = 0.30: 77%); we therefore emphasize effect estimates with 95% confidence intervals and report the minimum effects detectable with 80% power (w = 0.31, corresponding to Cramér’s V = 0.22, for the chi-square test and |ρ| = 0.26 for Spearman correlations) to aid the interpretation of non-significant results.

**Figure 1:**
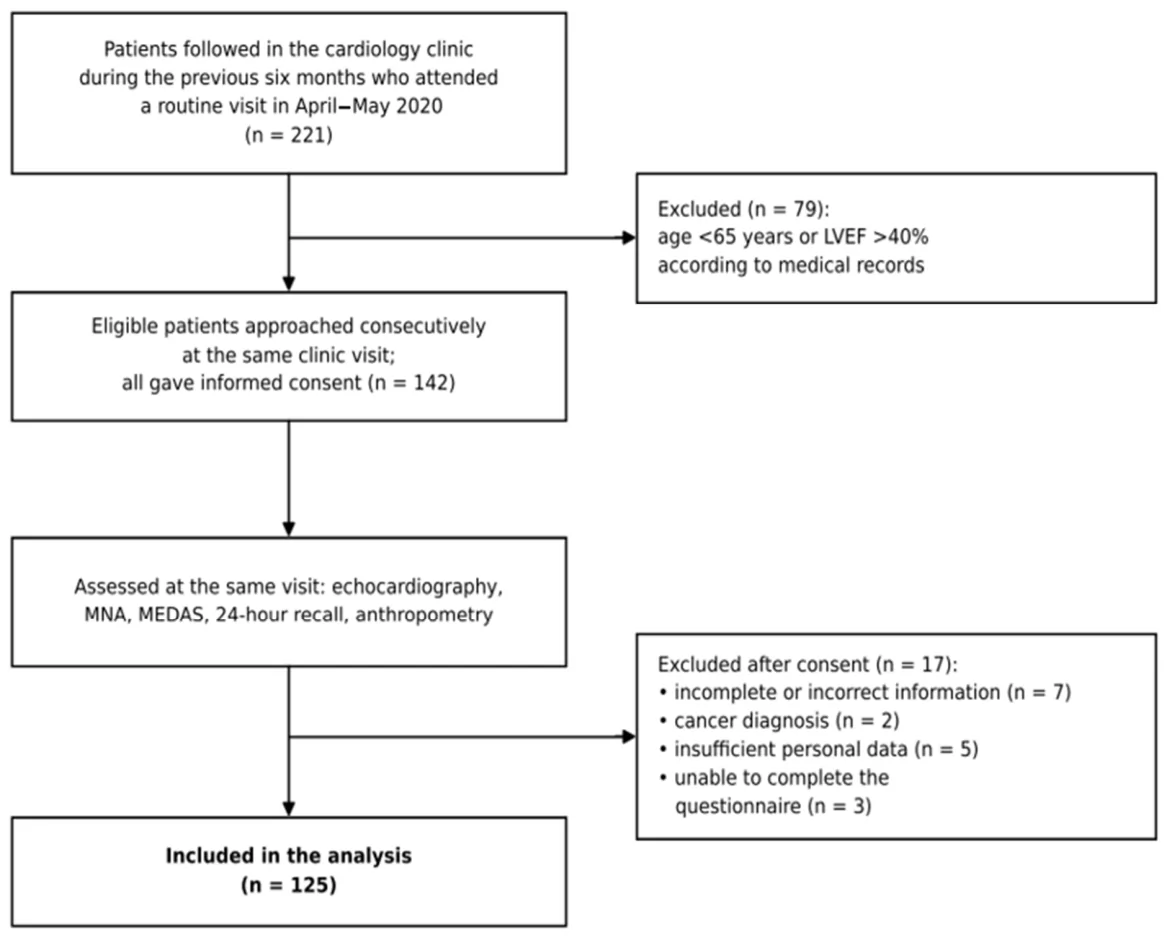
Flow diagram of participant selection. LVEF, left ventricular ejection fraction; MEDAS, Mediterranean Diet Adherence Screener; MNA, Mini Nutritional Assessment.

The study population consisted of 221 patients who had been followed in the cardiology clinic within the past six months and who attended the clinic in person for a routine visit during the study period. Of these, 79 patients did not meet the age or LVEF criteria according to their medical records. The remaining 142 eligible patients were approached consecutively at that visit, and all agreed to participate; recruitment and all assessments took place during this single routine visit, so no additional visit was required. Thus, the 142 eligible patients were those who attended the clinic in person during the recruitment period and did not include all eligible patients identified from previous medical records. Other patients followed in the clinic who met the eligibility criteria and whose follow-up visit fell within the study period but who did not attend, including any who deferred visits because of the pandemic restrictions, could not be approached; because eligibility was assessed at the clinic visit, their number was not recorded. After consent, 17 individuals were excluded: seven provided incomplete or incorrect information, two had a cancer diagnosis, five had insufficient personal data, and three were unable to complete the questionnaire. Ultimately, the study was completed with 125 participants, of whom 66.4% (*n* = 83) were male and 33.6% (*n* = 42) were female (Figure 1). All participants provided written and verbal informed consent prior to participation.

### 2.3. Anthropometric Measurements

Height was measured using a stadiometer (SECA, Hamburg, Germany) with a precision of 0.1 cm, with participants standing barefoot, heels together, and the head positioned in the Frankfurt plane. Body weight was measured using a calibrated digital scale (Tanita MC-580; Tanita Corporation, Tokyo, Japan), with participants wearing light indoor clothing and no shoes. Waist circumference was taken at the narrowest point between the lower costal (rib) border and the iliac crest, while hip circumference was measured at the level of the greatest gluteal protrusion using a flexible tape. All measurements were conducted in accordance with the International Society for the Advancement of Kinanthropometry (ISAK) standards for anthropometric assessment [21]. Waist-to-height ratio (WHtR), calculated as waist circumference (cm) divided by height (cm), was used as an indicator of central adiposity, with ≥0.60 as the predefined threshold for high central adiposity, consistent with current National Institute for Health and Care Excellence (NICE) guidance [22]; the more widely used threshold of ≥0.50 was additionally reported. Body mass index (BMI) was calculated as weight (kg)/height (m)^2^ and classified according to standard adult categories as underweight (<18.5 kg/m^2^), normal weight (18.5–24.9 kg/m^2^), overweight (25.0–29.9 kg/m^2^), obesity class I (30.0–34.9 kg/m^2^), obesity class II (35.0–39.9 kg/m^2^), and obesity class III (≥40.0 kg/m^2^) [23].

### 2.4. Clinical and Biochemical Data

Medical histories, medication use, and laboratory values were extracted from the electronic medical records system (PROBEL). LVEF was measured immediately before the nutritional interview so that cardiac and nutritional assessments were contemporaneous. HF etiology (ischemic cardiomyopathy), previous coronary angiography, percutaneous coronary intervention, coronary artery bypass grafting, an implantable cardioverter-defibrillator, and cardiac resynchronization therapy were recorded. Comorbidities including diabetes mellitus, hypertension, hyperlipidemia, chronic obstructive pulmonary disease (COPD), thyroid disorders, coronary artery disease, and chronic kidney disease (CKD) were documented as recorded diagnoses in the medical records. Because a recorded diagnosis may underestimate renal dysfunction, the estimated glomerular filtration rate (eGFR) was additionally calculated from serum creatinine using the 2021 race-free Chronic Kidney Disease Epidemiology Collaboration (CKD-EPI) equation [24] and categorized according to the Kidney Disease: Improving Global Outcomes (KDIGO) GFR categories [25]. Medications were recorded by class, including angiotensin-converting enzyme (ACE) inhibitors, beta-blockers, digitalis, diuretics, antiarrhythmics, anticoagulants, antihypertensives, statins, and antidiabetic agents; these classes were not mutually exclusive, and mineralocorticoid receptor antagonists, angiotensin receptor blockers, angiotensin receptor–neprilysin inhibitors, and diuretic subtypes were not recorded separately. New York Heart Association (NYHA) functional class, HF duration, atrial fibrillation, previous HF hospitalization, and natriuretic peptide levels were not available. Laboratory values were obtained from routine tests; tests requiring fasting (glucose and lipid profile) were performed in the fasting state, and missing laboratory values reflect tests that had not been ordered for every patient as part of routine care. Anemia was defined using the World Health Organization sex-specific hemoglobin thresholds (<13 g/dL for men and <12 g/dL for women) [26].

### 2.5. Dietary Assessment

A 24 h dietary recall was obtained from each participant through a face-to-face interview. Dietary intake was analyzed using BeBiS (Beslenme Bilgi Sistemleri, Nutrition Information Systems), version 8.2 (Ebispro for Windows, Stuttgart, Germany). Intakes were compared with the Estimated Average Requirement (EAR) [27]. For nutrients with an established EAR, we calculated the proportion of participants whose reported intake on the recall day was below their age- and sex-specific EAR, overall and separately for men and women. Because only one recall was available for each participant, within-person (day-to-day) variation could not be removed with a usual-intake model [27,28]; these proportions therefore describe reported intake on the recall day, do not estimate the prevalence of inadequate usual intake, and are presented as exploratory observations. Day-to-day variation widens the observed intake distribution, but the direction of any resulting difference from the prevalence of inadequate usual intake depends on the position of the EAR relative to the usual-intake distribution [27]. In addition, the requirement distribution for iron is skewed, so the EAR cut-point method is not strictly applicable to this nutrient, and the iron estimate should be interpreted with particular caution [27,29]. Where EAR values differed by age or sex, the corresponding age- and sex-specific reference values were applied, based on the relevant Dietary Reference Intakes [29,30,31,32]. For protein, the EAR was calculated using measured body weight and an EAR of 0.66 g/kg/day [33]; the higher intakes of 1.0 and 1.2 g/kg/day proposed for older adults were used as additional reference points [34]. For current smokers, the vitamin C EAR was adjusted by adding 35 mg/day to the age- and sex-specific reference value [32]. Vitamin A intake reported by BeBiS as retinol equivalents was converted to retinol activity equivalents before comparison with the corresponding DRI reference values [29]. Nutrients for which no EAR was established were compared descriptively with the Adequate Intake (AI): a group mean intake at or above the AI suggests a low prevalence of inadequate intake but does not establish adequacy, whereas a mean intake below the AI does not permit any conclusion about the prevalence of inadequacy [27]. Macronutrient intake was evaluated according to the Acceptable Macronutrient Distribution Ranges (AMDR) [33]; saturated fat, which has no AMDR, was compared with the dietary guideline recommendation of <10% of energy. Sodium was evaluated only against the Chronic Disease Risk Reduction (CDRR) level of 2300 mg/day [35]. Where applicable, Tolerable Upper Intake Levels (ULs) were also considered according to the relevant Dietary Reference Intakes [29,30,31,32,33]. Current use of dietary supplements (vitamin/mineral, protein, or fat-burning products) was recorded, but doses were not; therefore, supplement-derived intakes could not be quantified and were not included in the comparisons with reference values. The plausibility of reported energy intake was assessed as the ratio of energy intake to basal metabolic rate (EI:BMR), with BMR estimated using the Schofield equations [36]. Participants with an EI:BMR below the Goldberg cut-off for a single 24 h recall at the individual level (0.87, assuming a physical activity level of 1.55) were classified as under-reporters [37,38]; a physical activity level of 1.40 (cut-off 0.79) and the Mifflin–St Jeor equation [39] were used in sensitivity analyses.

### 2.6. Mini Nutritional Assessment (MNA)

The Mini Nutritional Assessment (MNA) is a validated, multidimensional screening and assessment tool for identifying nutritional risk and malnutrition in older adults. It includes anthropometric, dietary, functional, psychological, and illness-related items that yield a total score for nutritional-status classification [40]. The MNA has also been recommended in geriatric nutritional assessment by the International Association of Gerontology and Geriatrics (IAGG) and the International Academy of Nutrition and Aging (IANA) [41]. The full 18-item Turkish version of the MNA [42] was administered face-to-face by the researcher, a dietitian, according to the standard MNA instructions. The interviewer had not received separate formal training or certification in MNA administration. Item D (psychological stress or acute disease in the past three months) was scored on the basis of the participant’s report of acute illness or psychological stress; concerns related to the COVID-19 pandemic were not considered in scoring. Based on the total score, individuals are classified as having normal nutritional status (24–30 points), being at risk of malnutrition (17–23.5 points), or being malnourished (<17 points) [40]. The MNA is a screening and assessment instrument rather than a stand-alone diagnostic reference standard, and it does not apply the phenotypic and etiologic criteria of the Global Leadership Initiative on Malnutrition (GLIM) [43]; in this article, “malnourished” and “at risk of malnutrition” refer to MNA-defined categories.

### 2.7. Mediterranean Diet Adherence Screener (MEDAS)

The Mediterranean Diet Adherence Screener (MEDAS) was developed and validated for the PREDIMED study [44,45]. The Turkish version of the 14-item MEDAS, whose validity and reliability have been evaluated in Türkiye [46], was administered face-to-face, and the items were scored according to this adaptation [46]. Each item scores 1 point when its criterion is met and 0 otherwise: use of olive oil as the main culinary fat at least twice a week in salads or vegetable, meat, or fish dishes; ≥4 tablespoons of olive oil per day; ≥2 servings of vegetables per day; ≥3 servings of fruit per day; <100 g of red meat per day; <1 serving of butter or margarine per day; <1 serving (100 mL) of sugar-sweetened beverages per day; ≥7 glasses of wine per week; ≥3 servings of legumes per week; ≥3 servings of fish or seafood per week; <3 servings of commercial sweets or pastries per week; ≥3 servings of nuts per week; a higher consumption (by weight) of poultry, turkey, or rabbit than of red meat; and ≥2 servings per week of sofrito (a sauce of tomato, garlic, onion, or leek simmered with olive oil). In the original instrument, item 1 is scored only on whether olive oil is the main culinary fat and item 13 on the stated preference for white meat [44]; the Turkish adaptation applies a frequency criterion and an amount criterion to these items, respectively [46]. According to its developers, the frequency criterion for item 1 was introduced during the adaptation because the original wording was interpreted inconsistently by participants; thresholds of two, three, and four times per week were compared, and at least twice a week was adopted to make the item more concrete and standardized. Because item 1 differs from the original scoring, the main analyses were repeated after excluding it. The total score ranges from 0 to 14, with higher scores indicating greater adherence; item-level criteria, serving definitions, and responses are given in Supplementary Table S2. Participants were classified into three adherence levels: low (<7 points), moderate (7–8 points), and high (≥9 points). The threshold of ≥9 points corresponds to the PREDIMED definition of good adherence [44,45], and the additional cut-off at 7 points, which separates low from moderate adherence, follows the classification used in the Turkish adaptation and validation study of the MEDAS [46]; because the categorical analyses depend on these cut-offs, the main association was also analyzed with the continuous MEDAS score. No participant met the wine criterion, consistent with the low alcohol consumption customary among older adults of this sociocultural background in Türkiye; the item was retained to preserve the structure of the validated instrument. Because an item scored 0 by all participants does not alter any total score, its exclusion would leave all continuous analyses unchanged; the robustness of the categorical analyses to the cut-offs was examined in sensitivity analyses.

### 2.8. Statistical Analysis

Categorical variables were presented as frequencies and percentages, while continuous variables were summarized using means and standard deviations or medians and interquartile ranges, as appropriate. The normality of continuous variables was assessed using the Shapiro–Wilk test. All tests were two-sided, and analyses were based on available data without imputation; the number of participants with available data is reported for each variable. Associations between categorical variables were assessed using the Pearson chi-square test. When expected cell counts were small, the Fisher–Freeman–Halton test was estimated by Monte Carlo simulation. For continuous variables that did not meet the normality assumption, comparisons among more than two groups were performed using the Kruskal–Wallis H test. When the Kruskal–Wallis test was significant, pairwise comparisons were performed using Dunn’s test with Bonferroni correction.

The prespecified primary analysis assessed the association between MNA nutritional-status categories and MEDAS adherence categories using the Pearson chi-square test, and Cramér’s V was used to quantify the strength of the association. Adjusted standardized residuals were examined to describe the pattern of this association, using a Bonferroni-corrected threshold of |z| > 2.77 for the nine cells. The association between total MNA and MEDAS scores was also examined using Spearman rank correlation, and total MNA scores were compared across MEDAS adherence categories using the Kruskal–Wallis H test. Spearman rank correlation analyses were performed to assess the associations of continuous LVEF with total MNA and MEDAS scores. The 95% confidence intervals (CIs) of Spearman coefficients were calculated using Fisher’s z transformation with the Bonett–Wright standard error.

As a secondary analysis to account for potential confounding, ordinal logistic regression (proportional odds model) was used with MNA category (malnourished < at risk < normal) as the outcome and MEDAS score (per point) as the exposure; a separate model used MEDAS adherence categories (moderate and high vs. low). Covariates were prespecified on clinical grounds as potential confounders of the relationship between diet and nutritional status: age and sex, which influence both dietary habits and nutritional risk; education (≥high school), as a proxy for socioeconomic position and food access; diabetes mellitus and chronic kidney disease, which entail dietary prescriptions and can affect nutritional status; current smoking, which is associated with poorer diet quality; and diuretic use, as a marker of congestion that may affect the anthropometric MNA items. BMI was not included in the main model because it is a component of the MNA, but it was added in a sensitivity model, as was LVEF. The proportional odds assumption was assessed with a likelihood-ratio test against a multinomial model, convergence was confirmed for all models, multicollinearity was assessed with variance inflation factors and Spearman correlations among predictors, and effect modification by sex, diabetes, and diuretic use was tested with interaction terms, reported as ratios of odds ratios with 95% CIs. Because only 19 participants were classified as malnourished, the stability of the estimates was examined with nonparametric bootstrap resampling (2000 resamples; percentile 95% CIs) and with alternative specifications: an unadjusted model, a model adjusted only for age and sex, a model in which the recorded CKD diagnosis was replaced by eGFR <60 mL/min/1.73 m^2^ (*n* = 121), and binary logistic regression comparing normal nutritional status with the combined at-risk and malnourished categories. Linear regression with heteroskedasticity-robust (HC3) standard errors was used for continuous MNA scores.

Because some MNA items capture dietary intake that conceptually overlaps with MEDAS components, a sensitivity analysis was performed using modified MNA scores that excluded the protein-intake and fruit/vegetable items (K and L) and, in a broader model, all intake-related items (A, J, K, L, and M). Further sensitivity analyses were performed (i) stratified by diuretic use and using modified MNA scores excluding the volume-sensitive anthropometric items (BMI [F], mid-upper arm circumference [Q], and calf circumference [R]); (ii) after excluding energy under-reporters; (iii) after excluding current supplement users; (iv) with MNA item D (psychological stress or acute disease) set to “no” for all participants, which removes its contribution to the total score, together with a comparison of the association between participants with and without a positive item D; (v) with the MEDAS cut-offs proportionally rescaled to the 13 items that varied in this sample and, more conservatively, lowered by one point (low < 6, moderate 6–7, high ≥ 8); and (vi) after excluding MEDAS item 1, whose scoring in the Turkish adaptation differs from the original instrument, with the cut-offs rescaled proportionally to the 12 items that then varied (low < 6, moderate 6–7, high ≥ 8). Associations between individual MEDAS items and total MNA scores were examined with the Mann–Whitney U test (effect size r = z/√N), with Benjamini–Hochberg false discovery rate correction for multiple testing [47]. Hemoglobin and anemia were compared across MNA categories with the Kruskal–Wallis and chi-square tests, and the association between hemoglobin and total MNA score was examined with Spearman correlation and with linear regression adjusted for age, sex, and eGFR. Minimum detectable effect sizes at the achieved sample size were calculated for the chi-square test and for Spearman correlations (α = 0.05, power = 80%). Apart from the false discovery rate correction for the 13 MEDAS-item comparisons, secondary analyses were not adjusted for multiple comparisons; analyses of individual MEDAS items, of MEDAS adherence according to comorbidities, total cholesterol, and BMI categories, and of hemoglobin were considered exploratory. Statistical analyses were conducted using SPSS version 23.0 (IBM Corp., Armonk, NY, USA) and Python version 3.13.6 (Python Software Foundation, Beaverton, OR, USA); pandas 2.3.3 (NumFOCUS, Inc., Austin, TX, USA ), SciPy 1.17.1 (NumFOCUS, Inc., Austin, TX, USA ), and statsmodels 0.14.6 libraries (NumFOCUS, Inc., Austin, TX, USA ). Statistical significance was set at *p* < 0.05. Generative artificial intelligence (Claude Opus 5.5; Anthropic, San Francisco, CA, USA) was used to assist with data quality checks and statistical programming, including the supplementary sensitivity analyses. Only de-identified data were shared with the tool, in accordance with institutional data-governance requirements, and all analyses and code were checked independently by the authors.

## 3. Results

### 3.1. Participant Characteristics

A total of 125 participants (66.4% male) with a mean age of 72.9 ± 6.7 years were included (Figure 1); 64.0% were aged 65–74 years, 28.0% 75–84 years, and 8.0% ≥85 years (Table 1). All participants were diagnosed with heart failure. Ischemic cardiomyopathy was recorded in 34.4% (*n* = 43), and 52.8% (*n* = 66) had evidence of coronary artery disease when ischemic cardiomyopathy, a recorded coronary artery disease diagnosis, percutaneous coronary intervention, and coronary artery bypass grafting were considered together; the lower prevalence of a recorded coronary artery disease diagnosis (11.2%, *n* = 14) reflects how comorbidities were coded in the medical records rather than the prevalence of ischemic heart disease. In addition, 60% (*n* = 75) had hypertension, 44.8% (*n* = 56) diabetes mellitus, 19.2% (*n* = 24) hyperlipidemia, 15.2% (*n* = 19) COPD, 3.2% (*n* = 4) thyroid dysfunction, and 14.4% (*n* = 18) a recorded diagnosis of chronic kidney disease. ACE inhibitors were used by 40.8% (*n* = 51), beta-blockers by 36.8% (*n* = 46), diuretics by 56.8% (*n* = 71), and digitalis by 4.0% (*n* = 5); 36.8% (*n* = 46) used neither an ACE inhibitor nor a beta-blocker. Other medications included antihypertensives (80.8%, *n* = 101), antiarrhythmics (62.4%, *n* = 78), anticoagulants (62.4%, *n* = 78), non-cardiovascular drugs (48%, *n* = 60), antidiabetics (20.8%, *n* = 26), and statins (16.8%, *n* = 21). Only 10.4% (*n* = 13) reported prior nutrition education, mostly received from dietitians (92.3%), yet 92.8% (*n* = 116) had never followed a dietary treatment. Most participants (78%) consumed 2–3 meals per day, 60.8% (*n* = 76) reported never eating out, which is likely to reflect the stay-at-home restriction in force at the time, and 60.8% (*n* = 76) occasionally added extra salt to cooked meals.

### 3.2. Anthropometric, Biochemical, and Echocardiographic Findings

The mean BMI was 28.6 ± 6.0 kg/m^2^, and the mean waist circumference was 105.8 ± 18.5 cm. The mean waist-to-hip ratio was 1.01 ± 0.11, and the mean waist-to-height ratio (WHtR) was 0.64 ± 0.12. Overall, 63.2% of participants had a WHtR ≥ 0.60, and 86.4% had a WHtR ≥ 0.50, the threshold more commonly used in guidelines (Table 1).

Mean LVEF was 31.6 ± 6.3%, with values ranging from 15% to 40%. Among the available laboratory measurements, mean glucose was 148.8 ± 78.0 mg/dL, mean urea was 70.2 ± 39.9 mg/dL, and mean hemoglobin was 11.4 ± 2.1 g/dL (men 11.7 ± 2.3 g/dL; women 11.0 ± 1.6 g/dL). Using the sex-specific World Health Organization (WHO) thresholds, 69.1% (56/81) of participants with available hemoglobin had anemia (men 68.6%, women 70.0%). Mean eGFR was 60.8 ± 26.5 mL/min/1.73 m^2^, and 50.4% (61/121) of participants had an eGFR <60 mL/min/1.73 m^2^ (KDIGO categories G3a 18.2%, G3b 19.0%, G4 10.7%, and G5 2.5%), compared with 14.4% who had a recorded diagnosis of chronic kidney disease. Laboratory data were available for 70–123 participants because not all tests had been ordered for every patient as part of routine care. Biochemical and echocardiographic findings are presented in Table 2.

### 3.3. Dietary Intake and Nutrient Adequacy

Mean energy intake was 1604.8 ± 381.5 kcal/day. On the recall day, intake was below the EAR most frequently for vitamin D (98.4%), folate (86.4%), calcium (84.8%), vitamin B6 (72.0%), and vitamin A (69.6%), followed by vitamin C (59.2%) and zinc (53.6%). Intakes below the EAR were less common for vitamin E (30.4%), riboflavin (34.4%), vitamin B12 (28.8%), iodine (33.6%), iron (9.6%), and copper (6.4%). Proportions were similar in men and women for most nutrients; the larger differences for riboflavin (42.2% vs. 19.0%), zinc (61.4% vs. 38.1%), and calcium (80.7% vs. 92.9%) reflect the sex-specific EARs. Because these proportions are based on a single recall, they are exploratory observations rather than estimates of the prevalence of inadequate usual intake. One participant exceeded the UL for calcium; no participant exceeded the UL for the other nutrients assessed (Table 3). Mean protein intake was 0.84 ± 0.34 g/kg/day; recall-day intake was below the DRI EAR of 0.66 g/kg/day in 39.2% of participants and below the higher targets of 1.0 and 1.2 g/kg/day proposed for older adults in 75.2% and 84.8%, respectively (Table 3).

The mean EI:BMR was 1.08 ± 0.29, and 27 participants (21.6%) were classified as energy under-reporters (17.6% and 16.8% with the alternative definitions); none were over-reporters. Under-reporters did not differ from plausible reporters in age, sex, diabetes, MEDAS, or MNA scores. Among plausible reporters (*n* = 98), the proportion with intake below the EAR was lower for protein (27.6%), zinc (45.9%), and vitamin E (22.4%) but remained high for vitamin D (99.0%), folate (82.7%), calcium (82.7%), and vitamin A (69.4%). Eleven participants (8.8%) currently used dietary supplements; excluding them did not materially change these proportions (e.g., vitamin D 98.2%, folate 85.1%, calcium 83.3%) (Supplementary Table S3).

For nutrients without an established EAR, mean intakes of dietary fiber and potassium were below the AI in both sexes, so no conclusion about the prevalence of inadequacy can be drawn for these nutrients, whereas mean intakes of vitamin K, manganese, linoleic acid, and α-linolenic acid were at or above the AI in both sexes, suggesting a low prevalence of inadequate intake. Sodium intake was 3175.0 ± 1342.2 mg/day and exceeded the CDRR level of 2300 mg/day in 74.4% of participants (men 74.7%, women 73.8%) (Table 4).

The macronutrient distribution showed that total fat contributed 38.6 ± 8.3% of total energy, with 64.8% of participants above the upper AMDR boundary. Carbohydrate contributed 44.4 ± 8.6% of energy, with 55.2% of participants below the lower AMDR boundary. Saturated fat, which has no AMDR, contributed 13.0 ± 4.1% of energy, and 80.0% of participants exceeded the dietary guideline recommendation of <10% of energy. Linoleic acid and α-linolenic acid contributed 9.6 ± 4.4% and 0.9 ± 0.6% of energy, respectively (Table 5).

### 3.4. Nutritional Status and Mediterranean Diet Adherence

According to MNA screening items, 75.2% of participants reported no reduction in food intake, and only 10.4% reported >3 kg weight loss. Three individuals were non-mobile, and 60.0% reported psychological stress or acute disease in the past 3 months (item D). Neuropsychological status was generally good, and only 0.8% had a BMI < 19. In the assessment section, 3.2% had pressure ulcers, and 87.2% took more than three prescription drugs per day. For the MNA dietary components, 54.4% of participants met all three protein-intake criteria, while 58.4% consumed at least two servings of fruit or vegetables daily. Only 4% required assistance with eating, 28% perceived poor nutritional status, and 20% rated their health as good compared with peers. Overall, the MNA classified 15.2% of participants as malnourished, 49.6% as at risk of malnutrition, and 35.2% as having normal nutritional status. Nutritional risk frequently coexisted with excess adiposity: among the 81 participants classified as malnourished or at risk, 66.7% had a BMI ≥ 25 kg/m^2^, 33.3% a BMI ≥ 30 kg/m^2^, and 56.8% a WHtR ≥ 0.60. Detailed item-level MNA responses are provided in Supplementary Table S1.

Regarding individual MEDAS components, olive oil was used as the main cooking fat at least twice a week by 72.8% of participants, whereas adherence was lower for fish/seafood and nut consumption, with only 28.0% and 35.2% meeting the corresponding MEDAS criteria, respectively. At least three weekly servings of legumes were reported by 65.6% of participants, while 39.2% consumed at least three daily servings of fruit, and no participant met the wine criterion. Overall, 30.4% of participants had low adherence (<7), 43.2% had moderate adherence (7–8), and 26.4% had high adherence (≥9) (Table 6). Detailed item-level MEDAS responses are provided in Supplementary Table S2.

A statistically significant but modest association was observed between MNA nutritional-status categories and MEDAS adherence categories (χ^2^(4) = 11.26, *p* = 0.024; Cramér’s V = 0.21). Among participants classified as malnourished, 52.6% had low, 36.8% moderate, and 10.5% had high Mediterranean diet adherence. Among those at risk of malnutrition, 33.9% had low, 43.5% moderate, and 22.6% high adherence. Among participants with normal nutritional status, 15.9% had low, 45.5% moderate, and 38.6% high adherence (Table 6). Numerically, low adherence was more frequent in the malnourished group and high adherence in the group with normal nutritional status (adjusted standardized residuals +2.29 for low adherence among malnourished participants, and −2.60 for low and +2.29 for high adherence among those with normal nutritional status); however, no individual cell was statistically significant after Bonferroni correction (threshold |z| > 2.77).

Consistently, total MNA and MEDAS scores were positively correlated (ρ = 0.304, 95% CI 0.13–0.46, *p* < 0.001), and median MNA scores increased across low, moderate, and high MEDAS adherence (20.0 [16.6–22.9], 22.3 [19.1–25.0], and 24.0 [21.0–25.5], respectively; Kruskal–Wallis H = 11.17, *p* = 0.004). Post hoc Dunn–Bonferroni comparisons showed that MNA scores were lower in the low-adherence group than in the moderate-adherence (*p* = 0.046) and high-adherence groups (*p* = 0.004), whereas the moderate- and high-adherence groups did not differ (*p* = 0.746). In the sensitivity analysis, the association persisted after excluding MNA items K and L (ρ = 0.273, 95% CI 0.10–0.43, *p* = 0.002) and after excluding all intake-related items A, J, K, L, and M (ρ = 0.271, 95% CI 0.10–0.43, *p* = 0.002). In both sensitivity analyses, the difference between the low- and high-adherence groups remained significant after Bonferroni correction (*p* = 0.012 and *p* = 0.024, respectively).

In multivariable ordinal logistic regression, each one-point increase in MEDAS score was associated with higher odds of a better MNA category (adjusted odds ratio [OR] 1.40, 95% CI 1.15–1.72, *p* = 0.001; bootstrap 95% CI 1.18–1.80); the estimate was unchanged after additional adjustment for BMI or LVEF (Table 7A). In a separate model with MEDAS categories, the adjusted ORs compared with low adherence were 2.67 (95% CI 1.12–6.32; bootstrap 95% CI 1.10–7.91) for moderate and 4.68 (95% CI 1.71–12.78; bootstrap 95% CI 1.93–16.36) for high adherence (Table 7B); the wide intervals reflect the small number of malnourished participants. All models converged, variance inflation factors were ≤1.20, and the largest correlation between predictors was |ρ| = 0.29. The proportional odds assumption was met (*p* = 0.746), and there was no evidence of effect modification by sex (ratio of ORs 1.22, 95% CI 0.80–1.86), diabetes (0.96, 0.66–1.41), or diuretic use (0.82, 0.55–1.23). The estimate was similar without adjustment (OR 1.40, 95% CI 1.16–1.70), with adjustment for age and sex only (1.42, 1.17–1.72), when the recorded CKD diagnosis was replaced by eGFR < 60 mL/min/1.73 m^2^ (1.43, 1.16–1.77; *n* = 121), and in binary logistic regression comparing normal nutritional status with the at-risk and malnourished categories combined (1.36, 1.08–1.72) (Supplementary Table S7). For continuous MNA scores, each MEDAS point was associated with a 0.72-point higher score (95% CI 0.31–1.12) after adjustment, and with 0.58 (0.22–0.95) and 0.48 (0.17–0.78) points for the modified scores excluding dietary items.

The association was consistent in diuretic users (adjusted OR 1.35, 95% CI 1.03–1.75) and non-users (1.54, 1.08–2.18) and was unchanged when the volume-sensitive MNA items were excluded (ρ = 0.30, adjusted β = 0.68, *p* < 0.001). Few participants had low calf (*n* = 1) or mid-upper arm circumference (*n* = 0), so these measures could not be analyzed separately. The association also persisted after excluding energy under-reporters (ρ = 0.299; adjusted OR 1.43, 95% CI 1.12–1.81) and current supplement users (ρ = 0.306; adjusted OR 1.39, 95% CI 1.12–1.72). MNA item D was positive in 60.0% (*n* = 75) of participants, including all 19 participants classified as malnourished, and participants with a positive item D had lower MEDAS scores (median 7 [6,7,8] vs. 8 [7,8,9,10], *p* = 0.012). When item D was set to “no” for all participants, 7.2% were classified as malnourished, 47.2% as at risk, and 45.6% as having normal nutritional status; the continuous association was maintained (ρ = 0.294, 95% CI 0.12–0.45; adjusted OR 1.33, 95% CI 1.08–1.64), whereas the categorical association was no longer statistically significant (χ^2^(4) = 8.64, *p* = 0.071; Cramér’s V = 0.19). The association did not differ between participants with and without a positive item D (ratio of ORs 1.08, 95% CI 0.69–1.68) (Supplementary Table S5). Proportional rescaling of the MEDAS cut-offs to the 13 items that varied in this sample yielded identical adherence categories; with the cut-offs lowered by one point, the categorical association was weaker (χ^2^(4) = 8.27, *p* = 0.082), but high adherence remained associated with a better MNA category (adjusted OR 3.25, 95% CI 1.12–9.46). When MEDAS item 1 was excluded, the association was maintained (ρ = 0.299, 95% CI 0.13–0.45; adjusted OR per point 1.50, 95% CI 1.20–1.89), and with the cut-offs rescaled to the 12 items that varied, high adherence remained associated with a better MNA category (adjusted OR 4.28, 95% CI 1.61–11.39; χ^2^(4) = 10.04, *p* = 0.040) (Supplementary Table S6). In exploratory item-level analyses, meeting the MEDAS criteria for olive oil quantity, vegetables, fruit, and sofrito was associated with higher MNA scores after false discovery rate correction (median 23.5–24.0 vs. 20.0–20.5; all adjusted *p* ≤ 0.011), whereas fish, nut, and legume consumption were not (Supplementary Table S4).

Additional exploratory analyses showed no statistically significant associations between MEDAS adherence and total cholesterol or the presence of hypertension, diabetes mellitus, hyperlipidemia, COPD, thyroid disorders, coronary artery disease, or chronic kidney disease. MEDAS adherence did not differ significantly across the four BMI categories (Fisher–Freeman–Halton, Monte Carlo *p* = 0.10); when obesity classes I–III were analyzed separately, a borderline difference was observed (Monte Carlo *p* = 0.04), which should be interpreted cautiously given the small cell counts.

In exploratory analyses of the 81 participants with available hemoglobin, hemoglobin did not differ across MNA categories (11.1 ± 1.9, 11.3 ± 2.1, and 11.8 ± 2.3 g/dL in malnourished, at-risk, and normal participants; *p* = 0.671) and was not correlated with the total MNA score (ρ = 0.047, 95% CI −0.17 to 0.26; β adjusted for age, sex, and eGFR = 0.04 g/dL per MNA point, 95% CI −0.07 to 0.15; *n* = 79). The prevalence of anemia was numerically higher in the malnourished (81.8%) and at-risk (70.3%) groups than in those with normal nutritional status (63.6%), but the difference was not statistically significant (*p* = 0.517) (Supplementary Table S8). Participants with available hemoglobin had slightly higher MEDAS scores than those without (median 8 vs. 7, *p* = 0.027), whereas their MNA scores did not differ (*p* = 0.259).

### 3.5. Relationship Between LVEF and MNA and MEDAS Scores

To examine the relationship between resting systolic function and nutritional status or Mediterranean diet adherence, LVEF was analyzed as a continuous variable. Spearman rank correlation analysis showed no statistically significant correlation between EF and MNA total score (ρ = −0.145, 95% CI −0.31 to 0.03, *p* = 0.106) or between EF and MEDAS total score (ρ = 0.013, 95% CI −0.16 to 0.19, *p* = 0.885). These findings indicate that, within this cohort of older adults with heart failure and reduced ejection fraction, variation in resting EF was not significantly associated with nutritional status or Mediterranean diet adherence. However, with 125 participants, the study had 80% power only for correlations of |ρ| ≥ 0.26; therefore, weak associations cannot be excluded.

## 4. Discussion

The principal findings of this study were that higher MEDAS scores were modestly associated with better MNA classification, whereas neither MNA nor MEDAS scores were significantly associated with resting LVEF. In addition, recall-day intakes were below reference values for several micronutrients in a large proportion of participants, and protein, sodium, and macronutrient intakes frequently did not align with relevant dietary reference values.

The mean MEDAS score was 7.4 ± 1.9, and 26.4% of participants had high adherence. Higher Mediterranean diet adherence has been associated with lower mortality in older adults and lower incident HF risk [48,49], whereas evidence in established HF remains limited and inconsistent [18]. These studies give some context for the adherence observed in our cohort, but a cross-sectional design cannot show whether adherence conferred any clinical benefit.

The association between MNA and MEDAS was statistically significant but modest (Cramér’s V = 0.21; ρ = 0.30), and no individual cell remained significant after Bonferroni correction. The association with continuous MEDAS score remained after covariate adjustment (adjusted OR 1.40 per point), bootstrap resampling, and alternative model specifications. However, the two instruments overlap conceptually beyond their dietary items because the MNA also incorporates anthropometric, functional, illness-related, and self-perceived health domains that may relate to the ability to follow a Mediterranean dietary pattern. Excluding the dietary MNA items only slightly attenuated the correlation (ρ = 0.27 vs. 0.30) and did not eliminate this broader construct overlap. The cross-sectional design also rules out any conclusion about direction or causality: greater adherence may support better nutritional status, better-nourished or more functional patients may be better able to follow the diet, or both may reflect unmeasured socioeconomic, behavioral, functional, or disease-severity factors. Our data show an association between higher MEDAS scores and better MNA classification but do not show that Mediterranean diet adherence improves nutritional status. A recent study of 1072 community-dwelling older adults in Türkiye found no significant association between MEDAS and MNA Short-Form (MNA-SF) scores [50], suggesting that this relationship may vary by clinical context and population. MEDAS and MNA are best regarded as complementary rather than interchangeable measures.

Neither MNA score (*p* = 0.106) nor MEDAS score (*p* = 0.885) was correlated with resting LVEF. LVEF is a single measure of resting systolic performance and not an overall index of HF severity or cardiac function. The inclusion criterion also restricted LVEF to ≤40%, which narrowed its range and reduced the ability to detect correlations, and the study had 80% power only for correlations of |ρ| ≥ 0.26. Nutritional status and Mediterranean diet adherence were thus not associated with resting LVEF within this HFrEF cohort but this does not imply that they are unrelated to HF severity or prognosis. Nutritional risk assessed by MNA has been associated with adverse outcomes, including mortality, in patients with heart failure [51,52], and Mediterranean diet adherence has been investigated in relation to several clinical outcomes in heart failure [18,48]. Our findings regarding MEDAS and LVEF also differ from a recent cross-sectional study of 320 patients with heart failure, in which lower Mediterranean diet adherence was associated with lower LVEF [53]; differences in population characteristics, LVEF range, disease severity, and analytical methods may explain the different findings.

The MNA classified 15.2% of participants as malnourished and 49.6% as at risk of malnutrition, consistent with evidence that nutritional risk is common in heart failure and is associated with adverse clinical outcomes [51,52]. These figures should be distinguished from a formal diagnosis of malnutrition: the MNA is a screening and assessment tool, whereas the GLIM framework requires both phenotypic and etiologic criteria [43] and may identify a different subset of patients with HF [54]. In addition, 60.0% of our participants reported psychological stress or acute disease in the previous three months (MNA item D), and all participants classified as malnourished were in this group. Although pandemic-related concerns were not considered in scoring, the data were collected during the first wave of the COVID-19 pandemic, and when item D was neutralized, the proportion classified as malnourished fell from 15.2% to 7.2%. The MNA-based estimates may therefore overstate the burden of malnutrition in this population, and the categorical association with MEDAS should be read with this in mind; the continuous association, however, was preserved. Nutritional assessment remains clinically relevant even though MNA scores were not related to LVEF.

Nutritional risk coexisted with excess adiposity. Mean BMI was 28.6 kg/m^2^, 63.2% of participants had a WHtR ≥ 0.60, and two-thirds (66.7%) of those classified as malnourished or at risk had a BMI ≥ 25 kg/m^2^. This apparent paradox is consistent with the recognition that obesity and malnutrition are not mutually exclusive in HF [55]: BMI does not distinguish fat from lean mass, can be inflated by fluid retention, and may mask sarcopenic obesity. Because the BMI item contributes to the MNA score, a high BMI may also partly offset deficits in other domains, so that the MNA may under-detect nutritional risk in patients with obesity.

Anemia was also common: using sex-specific WHO thresholds, 69.1% of participants with available hemoglobin were anemic. Anemia is prognostically important in HF and is closely linked to iron deficiency and, less often, to deficiencies of folate and vitamin B12 [56]. Recall-day intakes were below the EAR for folate in 86.4% of participants but for vitamin B12 in only 28.8% and for iron in only 9.6%, and hemoglobin was not associated with MNA category or score. This pattern suggests that anemia in this cohort may reflect chronic disease, renal dysfunction, and impaired iron absorption or utilization more than dietary intake alone. Iron status and vitamin B12 and folate concentrations were not measured, however, and hemoglobin was available for only 81 participants.

Renal dysfunction was considerably more frequent than recorded diagnoses suggested: half of the participants had an eGFR < 60 mL/min/1.73 m^2^, whereas only 14.4% had a recorded diagnosis of CKD. CKD affects volume status and therefore anthropometric measurements, modifies dietary recommendations for protein, potassium, phosphorus, and sodium, and disturbs vitamin D and calcium–mineral metabolism [57]. The high proportions of participants with low recall-day intakes of vitamin D and calcium, and with protein intakes below 1.0–1.2 g/kg/day, should not be translated directly into dietary recommendations for individual patients with concomitant renal dysfunction, in whom nutritional targets need to be individualized. Replacing the recorded CKD diagnosis with eGFR < 60 mL/min/1.73 m^2^ in the regression model did not change the main association.

On the recall day, intake was below the EAR for vitamin D in 98.4% of participants, folate in 86.4%, calcium in 84.8%, vitamin B6 in 72.0%, and vitamin A in 69.6%, with substantial proportions also for vitamin C and zinc. Micronutrient deficiencies have frequently been described in patients with HF [58], but our data reflect reported intake rather than biochemical nutrient status. Moreover, the EAR cut-point method is designed to estimate the prevalence of inadequate usual intake from the distribution of usual intakes [27]. With a single 24 h recall, the observed distribution retains day-to-day variation and is wider than the usual-intake distribution, so the proportion below the EAR may differ from the prevalence of inadequate usual intake in either direction, depending on where the EAR lies relative to the usual-intake distribution [27,28]. In addition, the cut-point method assumes a symmetrical requirement distribution, an assumption that does not hold for iron, for which the probability approach is preferred [27,29]. The proportions reported here are therefore exploratory dietary signals rather than prevalence estimates. Systematic under-reporting, observed in about one-fifth of our participants, biases intakes downward; in sensitivity analyses, it affected some estimates, particularly protein, but not the high proportions for vitamin D, folate, calcium, and vitamin A, which persisted among plausible reporters. Absolute intakes may also have been influenced by the stay-at-home restriction in force at the time (see below). Technology-assisted approaches, such as image-based dietary records, may facilitate repeated, low-burden intake assessment in future studies of older adults with HF.

Protein intake also warrants attention. Although recall-day intake was below the DRI-based EAR of 0.66 g/kg/day in 39.2% of participants, 75.2% and 84.8% had intakes below 1.0 and 1.2 g/kg/day, respectively. These higher thresholds have been proposed in the context of older-adult nutritional needs and provide additional clinical context, but they should not be interpreted as equivalent to the DRI EAR [34]. Mean sodium intake was 3175.0 ± 1342.2 mg/day, and 74.4% of participants exceeded the CDRR level of 2300 mg/day. Evidence regarding sodium restriction in heart failure remains mixed: a systematic review and meta-analysis of randomized clinical trials did not demonstrate clear reductions in mortality or hospitalization, although potential effects on symptoms and quality of life have been reported [59]. Therefore, the present findings support attention to sodium intake while emphasizing individualized dietary assessment rather than a uniform dietary prescription.

The macronutrient profile also suggested several areas for dietary improvement. Total fat contributed 38.6 ± 8.3% of energy, with 64.8% of participants exceeding the upper AMDR boundary, while saturated fat contributed 13.0 ± 4.1% of energy and 80.0% of participants exceeded the dietary guideline recommendation of <10% of energy from saturated fat. These findings, together with low fiber intake, indicate that dietary quality was suboptimal in several domains beyond Mediterranean diet adherence alone. Certain components of the Mediterranean diet were relatively well established in this population, particularly olive oil use (72.8%), whereas fish and nut consumption were less frequent (28.0% and 35.2%, respectively). No participant met the MEDAS wine criterion, reflecting the low alcohol consumption customary among older adults of this sociocultural background in Türkiye; in such populations the MEDAS has an effective maximum of 13 points, which should be considered when adherence levels are compared with those of Mediterranean populations in which moderate wine consumption is common. This pattern suggests partial adherence to the Mediterranean dietary pattern and supports the relevance of culturally adapted dietary approaches in Türkiye [60].

Recruitment took place in April and May 2020, during the first wave of the COVID-19 pandemic and a stay-at-home restriction for adults aged ≥ 65 years in Türkiye. These conditions may have affected food access and eating behavior, so the MEDAS scores and the 24 h recall may reflect intake during the restriction rather than habitual intake; the high proportion of participants who never ate out (60.8%) probably also reflects this context. The pandemic may also have affected participant selection because only patients attending the clinic in person could be recruited. These factors limit the generalizability of the dietary and nutritional findings.

### 4.1. Implications for Health-Care Practice

These findings have practical implications for the organization of outpatient HF care. Nearly two-thirds of the participants were classified by the MNA as malnourished or at risk of malnutrition, yet only one in ten had ever received nutrition education, and fewer than one in ten had followed a dietary treatment. Because nutritional status was not associated with resting LVEF in this HFrEF cohort, LVEF should not be used as a proxy for nutritional risk. A validated nutritional screening instrument such as the MNA could be administered during routine outpatient visits and could be used to trigger referral to a dietitian within a multidisciplinary HF team [7]. The MEDAS serves a different purpose: it does not screen for malnutrition but characterizes diet quality, and it may complement nutritional screening by identifying modifiable dietary targets. The dietary patterns observed here, particularly low recall-day intakes of vitamin D, calcium, folate, and protein together with high sodium and saturated fat intake, indicate areas that may warrant individualized assessment and counseling, taking renal function and other comorbidities into account. Whether such screening and referral pathways improve clinical outcomes should be tested in prospective studies.

### 4.2. Strengths and Limitations

A major strength of this study is the combined assessment of nutritional status, Mediterranean diet adherence, and detailed dietary intake in consecutively recruited older adults with HFrEF, together with extensive sensitivity analyses. Nevertheless, several limitations should be considered. The cross-sectional design precludes causal inference and prevents assessment of the direction of the relationships between dietary factors, nutritional status, and cardiac function. The single-center sample, recruited during the COVID-19 stay-at-home restriction for older adults, limits generalizability. The final sample size was slightly below the prespecified target of 132 participants, which was based on the primary chi-square analysis; the regression analyses were secondary and not specifically powered, and the small number of malnourished participants (*n* = 19) resulted in wide confidence intervals for the categorical estimates. The study had 80% power only for correlations of |ρ| ≥ 0.26, so weak associations, including those with LVEF, cannot be excluded, and the restriction of LVEF to ≤40% may also have attenuated correlations with LVEF.

In addition, dietary intake was assessed using a single 24 h recall, so within-person variation could not be removed, and the proportions of participants whose reported intake on the recall day was below the EAR cannot establish the prevalence of inadequate usual intake; about one-fifth of participants were classified as energy under-reporters, and supplement doses were not recorded. HF was characterized from medical records, and NYHA functional class, HF duration, atrial fibrillation, previous HF hospitalization, natriuretic peptides, and the use of mineralocorticoid receptor antagonists, angiotensin receptor blockers, and angiotensin receptor–neprilysin inhibitors were not available; therefore, the available medication data do not permit a complete assessment of guideline-directed medical therapy. Recorded comorbidity diagnoses, particularly CKD, appeared to underestimate disease burden. eGFR was calculated from a single creatinine measurement, so chronicity could not be established and an eGFR < 60 mL/min/1.73 m^2^ should not be taken as a diagnosis of CKD. Residual confounding by HF severity, functional status, and socioeconomic factors cannot be excluded. LVEF was measured by a single cardiologist with a standardized protocol, but values were recorded rounded to the nearest 5%, as is usual in clinical practice, which limited the resolution of LVEF as a continuous variable, and the interviewer was not blinded to LVEF. MNA item D was positive in most participants during the pandemic period, which may have inflated MNA-defined nutritional risk. Hemoglobin and lipid measurements were available only for subsets of participants, and participants with available hemoglobin had slightly higher MEDAS scores. Edema and volume status, which may affect anthropometric measurements and MNA scores, were not assessed; this was only partly addressed by sensitivity analyses excluding the volume-sensitive MNA items and stratifying by diuretic use. As discussed above, data were collected in April–May 2020, during the stay-at-home restriction for adults aged ≥65 years, which may have influenced dietary habits, adherence levels, psychological stress, and participant selection. MEDAS item 1 was scored according to the Turkish adaptation, which uses a frequency criterion for olive oil use, although the results were similar when this item was excluded. Finally, the study was conducted in Türkiye, where dietary patterns, including the near absence of wine consumption among older adults, differ from those of other Mediterranean populations, which may limit the direct generalization of the findings.

## 5. Conclusions

In this cohort of older adults with HFrEF, nutritional risk identified by the MNA was common, and recall-day intakes of several micronutrients were frequently below reference values. Higher MEDAS scores were modestly associated with better MNA classification, whereas neither MEDAS nor MNA was associated with resting LVEF. The categorical association was attenuated and no longer statistically significant when MNA item D was neutralized, whereas the associations based on continuous scores persisted. Because the design was cross-sectional and the data were collected under pandemic-related restrictions, the direction of the association between Mediterranean diet adherence and nutritional status cannot be determined, and the absolute estimates may not reflect habitual intake. These findings suggest that dietary pattern and nutritional status provide complementary information that is not reflected by resting LVEF, and that micronutrient, protein, sodium, and macronutrient intakes may warrant individualized assessment. Prospective studies with repeated dietary assessments are needed to determine whether improving dietary adherence and nutritional status translates into better clinical outcomes.

## Figures and Tables

**Table 1:** Demographic, clinical, and anthropometric characteristics of the participants (*n* = 125).

Variable	*n*	Mean ± SD or *n* (%)	Median (Min–Max)
A. Demographic and clinical characteristics			
Age, years	125	72.9 ± 6.7	71 (65–90)
65–74 years		80 (64.0)	
75–84 years		35 (28.0)	
≥85 years		10 (8.0)	
Female sex	125	42 (33.6)	
Education	125		
Literate, no formal schooling		36 (28.8)	
Primary school		20 (16.0)	
High school		60 (48.0)	
University or higher		9 (7.2)	
Smoking status	125		
Current		23 (18.4)	
Former		46 (36.8)	
Never		56 (44.8)	
LVEF, %	125	31.6 ± 6.3	30 (15–40)
Ischemic cardiomyopathy	125	43 (34.4)	
Any evidence of coronary artery disease †	125	66 (52.8)	
Previous coronary angiography	125	44 (35.2)	
Percutaneous coronary intervention (stent)	125	18 (14.4)	
Coronary artery bypass grafting	125	10 (8.0)	
Implantable cardioverter-defibrillator	125	19 (15.2)	
Cardiac resynchronization therapy	125	0 (0.0)	
Comorbidities (recorded diagnoses)	125		
Hypertension		75 (60.0)	
Diabetes mellitus		56 (44.8)	
Hyperlipidemia		24 (19.2)	
COPD		19 (15.2)	
Thyroid disorder		4 (3.2)	
Coronary artery disease		14 (11.2)	
Chronic kidney disease		18 (14.4)	
eGFR, mL/min/1.73 m^2^ ‡	121	60.8 ± 26.5	59.6 (11.2–105.4)
eGFR < 60 mL/min/1.73 m^2^	121	61 (50.4)	
Medications §	125		
ACE inhibitor		51 (40.8)	
Beta-blocker		46 (36.8)	
Neither ACE inhibitor nor beta-blocker		46 (36.8)	
Diuretic		71 (56.8)	
Digitalis		5 (4.0)	
Antiarrhythmic		78 (62.4)	
Anticoagulant		78 (62.4)	
Antihypertensive		101 (80.8)	
Statin		21 (16.8)	
Antidiabetic		26 (20.8)	
Current dietary supplement use	125	11 (8.8)	
B. Anthropometric characteristics			
Body weight (kg)	125	78.55 ± 16.00	78.00 (45.00–149.00)
Height (cm)	125	165.98 ± 8.28	167.00 (145.00–186.00)
BMI (kg/m^2^)	125	28.59 ± 6.01	26.99 (16.53–58.20)
Waist circumference (cm)	125	105.83 ± 18.46	107.00 (69.00–155.00)
Hip circumference (cm)	125	104.79 ± 13.29	104.00 (78.00–140.00)
Waist-to-hip ratio	125	1.01 ± 0.11	1.03 (0.69–1.25)
Waist-to-height ratio	125	0.64 ± 0.12	0.63 (0.42–0.98)
WHtR ≥ 0.60	125	79 (63.2)	
WHtR ≥ 0.50	125	108 (86.4)	
Ideal body weight (kg)	125	61.31 ± 6.54	62.75 (47.50–77.00)

Data are *n* (%), or mean ± SD and median (min–max); percentages are based on participants with available data. † Ischemic cardiomyopathy, recorded coronary artery disease diagnosis, percutaneous coronary intervention, or coronary artery bypass grafting. ‡ Calculated with the 2021 race-free CKD-EPI creatinine equation; creatinine was not available for 4 participants. § Medication classes are not mutually exclusive; 45 of the 46 beta-blocker users were also recorded as receiving antiarrhythmic medication. NYHA functional class, HF duration, atrial fibrillation, previous HF hospitalization, natriuretic peptides, and use of mineralocorticoid receptor antagonists, angiotensin receptor blockers, or angiotensin receptor–neprilysin inhibitors were not available. Abbreviations: ACE, angiotensin-converting enzyme; BMI, body mass index; COPD, chronic obstructive pulmonary disease; eGFR, estimated glomerular filtration rate; LVEF, left ventricular ejection fraction; SD, standard deviation; WHtR, waist-to-height ratio.

**Table 2:** Biochemical and conventional echocardiographic findings.

Parameter	*n*	Mean ± SD	Median	Min–Max
Glu (mg/dL)	120	148.8 ± 78.0	121.0	69–513
Urea (mg/dL)	123	70.2 ± 39.9	55.6	12.5–210
Cr (mg/dL)	121	1.4 ± 0.8	1.13	0.57–4.92
eGFR (mL/min/1.73 m^2^)	121	60.8 ± 26.5	59.6	11.2–105.4
AST (U/L)	112	30.8 ± 28.8	22.0	9–165
ALT (U/L)	113	34.6 ± 56.8	16.0	5–283
TC (mg/dL)	73	182.4 ± 54.7	167.0	98–346
TG (mg/dL)	72	120.7 ± 65.2	111.0	51–392
LDL (mg/dL)	72	115.9 ± 43.5	105.5	42–224
HDL (mg/dL)	70	43.1 ± 13.5	42.0	18–83
HGB (g/dL), all	81	11.4 ± 2.1	11.6	6.9–16.8
Men	51	11.7 ± 2.3	12.3	6.9–16.8
Women	30	11.0 ± 1.6	11.1	7.8–14.3
Anemia, *n* (%) ‡	81	56 (69.1)		
LVEDD (cm)	116	5.63 ± 0.90	5.70	4.20–8.10
LVESD (cm)	116	4.03 ± 1.15	4.00	2.30–6.90
IVS (cm)	115	1.07 ± 0.17	1.00	0.70–1.50
PW (cm)	109	1.06 ± 0.14	1.00	0.70–1.40
EF (%)	125	31.61 ± 6.27	30.00	15–40

Glucose and lipid measurements were obtained in the fasting state. Numbers of participants differ because not all laboratory tests had been ordered for every patient as part of routine care and because some echocardiographic measurements were not recorded. Categories based on single laboratory reference ranges are not reported because such ranges are not appropriate for all participants (e.g., sex-specific hemoglobin thresholds and risk-based lipid targets in patients with established cardiovascular disease). eGFR was calculated with the 2021 race-free CKD-EPI creatinine equation. ‡ WHO thresholds: hemoglobin <13 g/dL in men and <12 g/dL in women; anemia was present in 35 of 51 men (68.6%) and 21 of 30 women (70.0%). Abbreviations: Glu, glucose; Cr, creatinine; eGFR, estimated glomerular filtration rate; AST, aspartate aminotransferase; ALT, alanine aminotransferase; TC, total cholesterol; TG, triglycerides; LDL, low-density lipoprotein; HDL, high-density lipoprotein; HGB, hemoglobin; LVEDD, left ventricular end-diastolic diameter; LVESD, left ventricular end-systolic diameter; IVS, interventricular septum thickness; PW, posterior wall thickness; EF, ejection fraction; SD, standard deviation.

**Table 3:** Daily nutrient intakes and proportion of participants whose recall-day intake was below the EAR (*n* = 125).

Nutrient (Unit)	Mean ± SD	Median (IQR)	EAR	% < EAR, All	Men (*n* = 83)	Women (*n* = 42)
Protein (g/day)	63.9 ± 23.4	60.8 (45.4–78.1)	0.66 g/kg/day	39.2	38.6	40.5
Carbohydrate (g/day)	179.6 ± 60.8	172.8 (137.0–210.9)	100 g/day	7.2	6.0	9.5
Vitamin A (µg RAE/day)	577.0 ± 417.3	436.3 (338.3–632.7)	625 (M)/500 (F)	69.6	71.1	66.7
Vitamin D (µg/day)	1.6 ± 3.5	0.6 (0.2–2.1)	10	98.4	98.8	97.6
Vitamin E (mg α-TE/day)	18.0 ± 9.7	16.4 (10.8–22.9)	12	30.4	30.1	31.0
Riboflavin (mg/day)	1.2 ± 0.4	1.1 (0.9–1.4)	1.1 (M)/0.9 (F)	34.4	42.2	19.0
Vitamin B6 (mg/day)	1.1 ± 0.4	1.1 (0.9–1.4)	1.4 (M)/1.3 (F)	72.0	72.3	71.4
Folate (µg DFE/day)	228.5 ± 72.0	223.3 (174.8–275.2)	320	86.4	84.3	90.5
Vitamin B12 (µg/day)	3.3 ± 2.0	2.7 (1.9–4.4)	2.0	28.8	27.7	31.0
Vitamin C (mg/day)	75.4 ± 60.1	59.6 (33.4–105.7)	75 (M)/60 (F); +35 smokers	59.2	62.7	52.4
Calcium (mg/day)	678.2 ± 334.1	624.6 (496.6–813.6)	800 (M 65–70)/1000 (M ≥71, F)	84.8	80.7	92.9
Iron (mg/day) *	9.2 ± 3.3	9.1 (6.5–10.9)	6 (M)/5 (F)	9.6	8.4	11.9
Zinc (mg/day)	8.6 ± 3.1	8.1 (6.2–10.2)	9.4 (M)/6.8 (F)	53.6	61.4	38.1
Copper (mg/day)	1.3 ± 0.5	1.2 (0.9–1.5)	0.7	6.4	4.8	9.5
Iodine (µg/day)	124.4 ± 59.4	117.7 (85.3–159.1)	95	33.6	36.1	28.6

Proportions refer to participants whose reported intake on the recall day was below the EAR. Because within-person variation could not be removed, they are exploratory observations and cannot establish the prevalence of inadequate usual intake; they do not indicate biochemical deficiency. * The estimate for iron is approximate because the iron requirement distribution is skewed. The calcium EAR is 800 mg/day for men aged 65–70 years and 1000 mg/day for men aged ≥ 71 years and all women. Vitamin A intakes were converted from retinol equivalents to RAE before comparison. The vitamin C EAR includes the additional 35 mg/day requirement for current smokers. EAR values were based on the corresponding age- and sex-specific Dietary Reference Intakes. Abbreviations: EAR, Estimated Average Requirement; IQR, interquartile range; RAE, retinol activity equivalents; DFE, dietary folate equivalents; α-TE, α-tocopherol equivalents.

**Table 4:** Intakes of nutrients without an established EAR, and sodium intake relative to the CDRR (*n* = 125).

Nutrient (Unit)	Mean ± SD	Median (IQR)	AI, Men/Women	Mean, Men/Women	Mean Relative to AI ‡
A. Nutrients with an Adequate Intake					
Dietary fiber (g/day)	18.4 ± 7.3	17.5 (13.3–23.9)	30/21	19.2/16.9	Below/below
Vitamin K (µg/day)	282.0 ± 190.6	217.7 (148.2–369.8)	120/90	301.2/244.1	At or above/at or above
Potassium (mg/day)	1954.0 ± 746.4	1921.0 (1395.4–2456.1)	3400/2600	1998.1/1866.8	Below/below
Manganese (mg/day)	3.0 ± 1.3	2.8 (2.2–3.6)	2.3/1.8	3.0/2.9	At or above/at or above
Linoleic acid, *n*-6 (g/day)	17.2 ± 9.5	15.8 (10.1–21.8)	14/11	17.1/17.5	At or above/at or above
α-Linolenic acid, *n*-3 (g/day)	1.6 ± 1.4	1.3 (0.9–1.8)	1.6/1.1	1.6/1.5	At or above/at or above
B. Sodium relative to the Chronic Disease Risk Reduction level					
Sodium (mg/day)	3175.0 ± 1342.2	2925.3 (2299.8–3769.3)	CDRR 2300	3226.2/3073.9	Above CDRR: 74.4% (men 74.7%, women 73.8%)

‡ A group mean intake at or above the AI implies a low prevalence of inadequate intake but does not establish adequacy for individuals; a group mean below the AI does not permit conclusions about the prevalence of inadequacy [27]. AI values were based on the corresponding age- and sex-specific Dietary Reference Intakes. Sodium is evaluated only against the Chronic Disease Risk Reduction (CDRR) level of 2300 mg/day. Abbreviations: AI, Adequate Intake; CDRR, Chronic Disease Risk Reduction level; IQR, interquartile range.

**Table 5:** Contribution of macronutrients to total energy relative to the AMDR and, for saturated fat, the dietary guideline recommendation (*n* = 125).

Macronutrient	% of Energy, Mean ± SD	Reference (% of Energy)	% Below	% Within	% Above
A. Acceptable Macronutrient Distribution Range					
Protein	16.0 ± 4.5	10–35	3.2	96.8	0.0
Total fat	38.6 ± 8.3	20–35	2.4	32.8	64.8
Carbohydrate	44.4 ± 8.6	45–65	55.2	42.4	2.4
Linoleic acid (*n*-6)	9.6 ± 4.4	5–10	17.6	40.8	41.6
α-Linolenic acid (*n*-3)	0.9 ± 0.6	0.6–1.2	33.6	50.4	16.0
B. Dietary guideline recommendation (no AMDR)					
Saturated fat	13.0 ± 4.1	<10 (guideline)	—	20.0	80.0

AMDR values were based on the Dietary Reference Intakes for macronutrients. Saturated fat has no AMDR and is shown separately (panel B) against the dietary guideline recommendation of <10% of energy (“within”, <10%; “above”, ≥10%). Abbreviations: AMDR, Acceptable Macronutrient Distribution Range.

**Table 6:** MNA nutritional status and MEDAS adherence categories and their association (*n* = 125).

MNA Nutritional Status	*n* (%)	MEDAS Low (<7), *n* (%)	MEDAS Moderate (7–8), *n* (%)	MEDAS High (≥9), *n* (%)
Malnourished	19 (15.2)	10 (52.6)	7 (36.8)	2 (10.5)
At risk of malnutrition	62 (49.6)	21 (33.9)	27 (43.5)	14 (22.6)
Normal nutritional status	44 (35.2)	7 (15.9)	20 (45.5)	17 (38.6)
Total	125 (100.0)	38 (30.4)	54 (43.2)	33 (26.4)
Association statistics	χ^2^(4) = 11.26	*p* = 0.024	Cramér’s V = 0.21	

Abbreviations: MNA, Mini Nutritional Assessment; MEDAS, Mediterranean Diet Adherence Screener. Data are presented as *n* (row %). The association was assessed using the Pearson chi-square test, and Cramér’s V was used to quantify its strength. No individual cell was statistically significant after Bonferroni correction of the adjusted standardized residuals.

**Table 7:** Multivariable ordinal logistic regression for a better MNA nutritional-status category: (A) continuous MEDAS score and (B) MEDAS adherence categories (*n* = 125).

Variable	Adjusted OR	95% CI	Bootstrap 95% CI †	*p*
A. Model 1: MEDAS score (continuous)				
MEDAS score (per 1 point)	1.40	1.15–1.72	1.18–1.80	0.001
Age (years)	0.97	0.92–1.02	—	0.227
Female sex	0.85	0.39–1.86	—	0.681
Education ≥ high school	1.12	0.53–2.37	—	0.757
Diabetes mellitus	1.27	0.63–2.60	—	0.504
Chronic kidney disease	1.05	0.38–2.92	—	0.928
Current smoking	0.79	0.30–2.05	—	0.623
Diuretic use	0.71	0.35–1.44	—	0.347
B. Model 2: MEDAS adherence categories (reference: low adherence)				
Moderate vs. low adherence	2.67	1.12–6.32	1.10–7.91	0.026
High vs. low adherence	4.68	1.71–12.78	1.93–16.36	0.003
Age (years)	0.97	0.92–1.02	—	0.268
Female sex	0.88	0.40–1.92	—	0.741
Education ≥ high school	1.06	0.50–2.25	—	0.881
Diabetes mellitus	1.21	0.59–2.46	—	0.606
Chronic kidney disease	0.95	0.34–2.67	—	0.929
Current smoking	0.80	0.31–2.09	—	0.653
Diuretic use	0.65	0.32–1.33	—	0.239

Panels A and B are two separate models, each adjusted for all covariates listed in the panel. OR > 1 indicates higher odds of a better MNA category (malnourished < at risk < normal; *n* = 19, 62, and 44, respectively). † Nonparametric percentile bootstrap (2000 resamples; all models converged), shown for the exposure of interest. Proportional odds assumption (panel A): likelihood-ratio test *p* = 0.746. Variance inflation factors were ≤1.20. Additional model specifications are shown in Supplementary Table S7. Abbreviations: CI, confidence interval; MEDAS, Mediterranean Diet Adherence Screener; MNA, Mini Nutritional Assessment; OR, odds ratio.

## Data Availability

The data presented in this study are available on request from the corresponding author due to privacy and ethical restrictions.

## Supplementary Materials

The following supporting information can be downloaded at: https://www.mdpi.com/article/10.3390/healthcare14213675/s1, Table S1: Mini Nutritional Assessment (MNA) findings (*n* = 125); Table S2: Mediterranean Diet Adherence Screener (MEDAS): scoring criteria and findings (*n* = 125); Table S3: Energy intake plausibility, supplement use, and sensitivity of the proportions of participants with recall-day intake below the EAR (*n* = 125); Table S4: Total MNA scores according to individual MEDAS items (exploratory analysis; *n* = 125); Table S5: Sensitivity analysis for MNA item D (psychological stress or acute disease in the past 3 months); Table S6: Sensitivity of the MNA–MEDAS association to the MEDAS scoring and cut-offs; Table S7: Robustness of the ordinal logistic regression estimates; Table S8: Hemoglobin and anemia according to MNA nutritional-status category (participants with available hemoglobin, *n* = 81).

## Abbreviations

AI	Adequate Intake
AMDR	Acceptable Macronutrient Distribution Range
BMI	Body mass index
BMR	Basal metabolic rate
CDRR	Chronic Disease Risk Reduction
CI	Confidence interval
CKD	Chronic kidney disease
COPD	Chronic obstructive pulmonary disease
DRI	Dietary Reference Intake
EAR	Estimated Average Requirement
eGFR	Estimated glomerular filtration rate
EI	Energy intake
GLIM	Global Leadership Initiative on Malnutrition
HF	Heart failure
HFrEF	Heart failure with reduced ejection fraction
LVEF	Left ventricular ejection fraction
MedDiet	Mediterranean diet
MEDAS	Mediterranean Diet Adherence Screener
MNA	Mini Nutritional Assessment
NYHA	New York Heart Association
OR	Odds ratio
UL	Tolerable Upper Intake Level
WHO	World Health Organization
WHR	Waist-to-hip ratio
WHtR	Waist-to-height ratio
