# Supplementary material for: Mediterranean Diet Adherence and Nutritional Status in Older Adults with Heart Failure with Reduced Ejection Fraction: A Cross-Sectional Study
Source: Healthcare (Basel). 2026 Oct 30;14(21):3675. doi: 10.3390/healthcare14213675 (PMC13763292; doi:10.3390/healthcare14213675)
Supplement: Supplementary file 1 [file healthcare-14-03675-s001.zip › healthcare-4634586-supplementary.pdf]

**Table S1.** Mini Nutritional Assessment (MNA) findings (*n* = 125).

| Item | Response (points) | *n* | % |
|---|---|---|---|
| **Screening items** | | | |
| A. Decline in food intake over the past 3 months due to loss of appetite, digestive problems, or chewing or swallowing difficulties | Severe decrease (0) | 8 | 6.4 |
| | Moderate decrease (1) | 23 | 18.4 |
| | No decrease (2) | 94 | 75.2 |
| B. Weight loss during the past 3 months | >3 kg (0) | 13 | 10.4 |
| | Does not know (1) | 10 | 8.0 |
| | 1–3 kg (2) | 17 | 13.6 |
| | No weight loss (3) | 85 | 68.0 |
| C. Mobility | Bed or chair bound (0) | 3 | 2.4 |
| | Gets out of bed/chair but does not go out (1) | 58 | 46.4 |
| | Goes out (2) | 64 | 51.2 |
| D. Psychological stress or acute disease in the past 3 months | Yes (0) | 75 | 60.0 |
| | No (2) | 50 | 40.0 |
| E. Neuropsychological problems | Severe dementia or depression (0) | 1 | 0.8 |
| | Mild dementia (1) | 4 | 3.2 |
| | No psychological problems (2) | 120 | 96.0 |
| F. Body mass index (kg/m$^2$) | <19 (0) | 1 | 0.8 |
| | 19 to <21 (1) | 3 | 2.4 |
| | 21 to <23 (2) | 11 | 8.8 |
| | ≥23 (3) | 110 | 88.0 |
| **Assessment items** | | | |
| G. Lives independently (not in a nursing home or hospital) | Yes (1) | 89 | 71.2 |
| | No (0) | 36 | 28.8 |
| H. Takes more than 3 prescription drugs per day | Yes (0) | 109 | 87.2 |
| | No (1) | 16 | 12.8 |
| I. Pressure sores or skin ulcers | Yes (0) | 4 | 3.2 |
| | No (1) | 121 | 96.8 |
| J. Number of full meals per day | 1 meal (0) | 6 | 4.8 |
| | 2 meals (1) | 39 | 31.2 |
| | 3 meals (2) | 80 | 64.0 |
| K. Protein intake markers (dairy; legumes or eggs; meat, fish, or poultry) | 0–1 yes (0.0) | 24 | 19.2 |
| | 2 yes (0.5) | 33 | 26.4 |
| | 3 yes (1.0) | 68 | 54.4 |
| L. ≥2 servings of fruit or vegetables per day | No (0) | 52 | 41.6 |
| | Yes (1) | 73 | 58.4 |
| M. Fluid intake per day (water, juice, coffee, tea, milk, etc.) | <3 cups (0.0) | 21 | 16.8 |
| | 3–5 cups (0.5) | 64 | 51.2 |
| | >5 cups (1.0) | 40 | 32.0 |
| N. Mode of feeding | Unable to eat without assistance (0) | 5 | 4.0 |
| | Self-fed with some difficulty (1) | 44 | 35.2 |
| | Self-fed without any problem (2) | 76 | 60.8 |
| O. Self-perception of nutritional status | Views self as malnourished (0) | 35 | 28.0 |
| | Uncertain (1) | 48 | 38.4 |
| | No nutritional problem (2) | 42 | 33.6 |
| P. Health status compared with peers | Not as good (0.0) | 40 | 32.0 |
| | Does not know (0.5) | 60 | 48.0 |
| | As good (1.0) | 25 | 20.0 |
| | Better (2.0) | 0 | 0.0 |

| Item | Response (points) | *n* | % |
|---|---|---|---|
| Q. Mid-upper arm circumference (cm) | <21 (0.0) | 0 | 0.0 |
| | 21–22 (0.5) | 3 | 2.4 |
| | >22 (1.0) | 122 | 97.6 |
| R. Calf circumference (cm) | <31 (0) | 1 | 0.8 |
| | ≥31 (1) | 124 | 99.2 |
| **MNA classification (total score)** | | | |
| <17 points | Malnourished | 19 | 15.2 |
| 17–23.5 points | At risk of malnutrition | 62 | 49.6 |
| 24–30 points | Normal nutritional status | 44 | 35.2 |

Data are presented as n and %. The full 18-item Turkish version of the MNA was used. Points for each response are shown in parentheses; category boundaries follow the MNA instructions (e.g., BMI 19 to <21 kg/m$^2$). Classification: <17 points, malnourished; 17–23.5 points, at risk of malnutrition; 24–30 points, normal nutritional status. Item D was scored on the basis of the participant's report of acute illness or psychological stress; pandemic-related concerns were not considered.

**Table S2.** Mediterranean Diet Adherence Screener (MEDAS): scoring criteria and findings (*n* = 125).

| MEDAS item (serving definition used in the Turkish form) | Criterion for 1 point | *n* (%) meeting criterion |
|---|---|---|
| 1. Use of olive oil as the main culinary fat (salads, vegetable, meat or fish dishes) | ≥2 times per week * | 91 (72.8) |
| 2. Olive oil per day, including frying, salads, and meals eaten away from home (1 tablespoon = 13.5 g) | ≥4 tablespoons | 60 (48.0) |
| 3. Vegetables per day (1 serving = 200 g) | ≥2 servings | 68 (54.4) |
| 4. Fruit, including freshly squeezed juice, per day (1 serving = 80 g fruit or 100 mL juice) | ≥3 servings | 49 (39.2) |
| 5. Red meat per day | <100 g | 80 (64.0) |
| 6. Butter or margarine per day (1 serving = 1 tablespoon, 12 g) | <1 serving | 80 (64.0) |
| 7. Sugar-sweetened or carbonated beverages per day (1 serving = 100 mL) | <1 serving | 87 (69.6) |
| 8. Wine per week (1 glass = 125 mL) | ≥7 glasses | 0 (0.0) |
| 9. Legumes per week (1 serving = 150 g) | ≥3 servings | 82 (65.6) |
| 10. Fish or seafood per week (1 serving = 100–150 g fish or 200 g shellfish) | ≥3 servings | 35 (28.0) |
| 11. Commercial (not homemade) sweets or pastries per week | <3 times | 93 (74.4) |
| 12. Nuts, including peanuts, per week (1 serving = 30 g) | ≥3 servings | 44 (35.2) |
| 13. Preference for chicken, turkey, or rabbit instead of beef, pork, hamburger, or sausage | White meat > red meat (by weight) * | 89 (71.2) |
| 14. Dishes with sofrito (tomato, garlic, onion, or leek sauce simmered with olive oil) per week | ≥2 times | 67 (53.6) |
| **Total score and adherence categories** | | |
| Total MEDAS score, mean ± SD | 0–14 | 7.4 ± 1.9 |
| Low adherence | Score <7 | 38 (30.4) |
| Moderate adherence | Score 7–8 | 54 (43.2) |
| High adherence | Score ≥9 | 33 (26.4) |

Items were scored according to the Turkish adaptation of the MEDAS (Pehlivanoğlu et al.); each item scores 1 point when the criterion is met and 0 otherwise, and the total score ranges from 0 to 14. * In the original instrument, item 1 is scored only on whether olive oil is the main culinary fat and item 13 on the stated preference for white meat; the Turkish adaptation applies the frequency and amount criteria shown. Results after excluding item 1 are given in Supplementary Table S6. No participant met the wine criterion, so the effective maximum score in this sample was 13.

**Table S3.** Energy intake plausibility, supplement use, and sensitivity of the proportions of participants with recall-day intake below the EAR (*n* = 125).

**A. Energy intake plausibility**

| Measure | Value |
| --- | --- |
| EI:BMR (Schofield), mean ± SD | 1.08 ± 0.29 |
| EI:BMR (Mifflin–St Jeor), mean ± SD | 1.16 ± 0.33 |
| Under-reporters, Schofield, PAL 1.55 (cut-off 0.87) | 27 (21.6%) |
| Under-reporters, Schofield, PAL 1.40 (cut-off 0.79) | 22 (17.6%) |
| Under-reporters, Mifflin–St Jeor, PAL 1.55 | 21 (16.8%) |
| Over-reporters (cut-off 2.75) | 0 |
| Energy intake, plausible reporters (n = 98), mean ± SD | 1733.2 ± 312.1 kcal/day |
| Current supplement users | 11 (8.8%): vitamin/mineral 7, protein 3, fat-burning 1 |

**B. Proportion of participants with recall-day intake below the EAR (%)**

| Nutrient | All (n = 125) | Plausible reporters (n = 98) | Supplement non-users (n = 114) |
| --- | --- | --- | --- |
| Vitamin D | 98.4 | 99.0 | 98.2 |
| Folate | 86.4 | 82.7 | 85.1 |
| Calcium | 84.8 | 82.7 | 83.3 |
| Vitamin B6 | 72.0 | 65.3 | 70.2 |
| Vitamin A | 69.6 | 69.4 | 68.4 |
| Vitamin C | 59.2 | 58.2 | 57.9 |
| Zinc | 53.6 | 45.9 | 51.8 |
| Protein (0.66 g/kg/day) | 39.2 | 27.6 | 40.4 |
| Riboflavin | 34.4 | 29.6 | 33.3 |
| Iodine | 33.6 | 32.7 | 32.5 |
| Vitamin E | 30.4 | 22.4 | 30.7 |
| Vitamin B12 | 28.8 | 28.6 | 30.7 |
| Iron * | 9.6 | 6.1 | 9.6 |
| Copper | 6.4 | 1.0 | 7.0 |

BMR, basal metabolic rate; EAR, Estimated Average Requirement; EI, energy intake; PAL, physical activity level. Under-reporting was defined using the Goldberg cut-off for a single 24-hour recall at the individual level. Proportions refer to participants whose reported intake on the recall day was below the EAR; because within-person variation could not be removed, they are exploratory observations and cannot establish the prevalence of inadequate usual intake. Supplement-derived intakes could not be quantified because doses were not recorded. * The iron estimate is approximate because the iron requirement distribution is skewed.

**Table S4.** Total MNA scores according to individual MEDAS items (exploratory analysis; $n = 125$).

| MEDAS item (criterion met) | n meeting | MNA median, met | MNA median, not met | Mean difference | r | *p* | Adjusted *p* * |
|---|---|---|---|---|---|---|---|
| Olive oil as main fat | 91 | 22.0 | 21.8 | 1.21 | 0.10 | 0.264 | 0.382 |
| Olive oil ≥4 tbsp/day | 60 | 23.5 | 20.5 | 2.82 | 0.32 | <0.001 | 0.002 |
| Vegetables ≥2 servings/day | 68 | 23.5 | 20.5 | 2.84 | 0.32 | <0.001 | 0.002 |
| Fruit ≥3 servings/day | 49 | 23.5 | 20.5 | 2.10 | 0.26 | 0.003 | 0.011 |
| Red meat <1 serving/day | 80 | 21.2 | 23.0 | −1.34 | −0.13 | 0.149 | 0.243 |
| Butter/margarine <1 serving/day | 80 | 21.5 | 22.5 | −0.55 | −0.06 | 0.494 | 0.583 |
| Sugary drinks <1/day | 87 | 21.5 | 23.0 | −1.58 | −0.17 | 0.055 | 0.131 |
| Legumes ≥3/week | 82 | 22.8 | 20.5 | 1.42 | 0.15 | 0.085 | 0.157 |
| Fish/seafood ≥3/week | 35 | 21.0 | 22.0 | 0.18 | −0.02 | 0.867 | 0.867 |
| Commercial sweets <3/week | 93 | 22.0 | 22.0 | −0.79 | −0.05 | 0.610 | 0.661 |
| Nuts ≥3/week | 44 | 23.0 | 21.5 | 1.69 | 0.17 | 0.060 | 0.131 |
| Prefers white meat | 89 | 21.5 | 23.0 | −0.98 | −0.09 | 0.293 | 0.382 |
| Sofrito ≥2/week | 67 | 24.0 | 20.0 | 2.75 | 0.32 | <0.001 | 0.002 |

Mann–Whitney U test; $r = z/\sqrt{N}$. * Benjamini–Hochberg false discovery rate correction for 13 tests. The wine item was not analyzed because no participant met the criterion.

**Table S5.** Sensitivity analysis for MNA item D (psychological stress or acute disease in the past 3 months).

| Measure | Full MNA | Item D set to "no" / Statistic |
|---|---|---|
| **A. Item D responses** | | |
| Positive item D (stress or acute disease), n (%) | 75 (60.0) | |
| Among malnourished / at risk / normal, n/N | 19/19 / 49/62 / 7/44 | |
| MEDAS score, median (IQR): item D positive vs. negative | 7 (6–8) vs. 8 (7–10) | *p* = 0.012 |
| **B. Main analyses with the full MNA and with item D set to "no" for all participants** | | |
| Malnourished, n (%) | 19 (15.2) | 9 (7.2) |
| At risk of malnutrition, n (%) | 62 (49.6) | 59 (47.2) |
| Normal nutritional status, n (%) | 44 (35.2) | 57 (45.6) |
| MNA × MEDAS categories, $\chi^2(4)$; p; Cramér's V | 11.26; 0.024; 0.21 | 8.64; 0.071; 0.19 † |
| Spearman ϱ, MNA vs. MEDAS score (95% CI) | 0.304 (0.13 to 0.46) | 0.294 (0.12 to 0.45) |
| Adjusted OR per MEDAS point (95% CI) ‡ | 1.40 (1.15–1.72) | 1.33 (1.08–1.64) |
| **C. Association according to item D** | | |
| Item D positive (n = 75): ϱ (95% CI); adjusted OR (95% CI) | 0.210 (−0.02 to 0.42) | 1.46 (1.07–1.99) |
| Item D negative (n = 50): ϱ (95% CI); adjusted OR (95% CI) | 0.230 (−0.06 to 0.48) | 1.25 (0.87–1.80) |
| Interaction MEDAS × item D: ratio of ORs (95% CI); p | 1.08 (0.69–1.68) | *p* = 0.744 |

Setting item D to "no" (2 points) for all participants removes the contribution of this item to the total score while preserving the standard MNA cut-offs. In panel C, the second and third columns give Spearman ϱ and the adjusted OR, respectively. † One expected cell count was <5 (minimum 2.4). ‡ Ordinal logistic regression adjusted for age, sex, education, diabetes mellitus, chronic kidney disease, current smoking, and diuretic use.

**Table S6.** Sensitivity of the MNA–MEDAS association to the MEDAS scoring and cut-offs.

| Measure | Full score, original cut-offs (<7, 7–8, ≥9) | Full score, rescaled to 13 items * | Full score, cut-offs lowered by one point (<6, 6–7, ≥8) | Item 1 excluded, rescaled to 12 items (<6, 6–7, ≥8) ‡ |
|---|---|---|---|---|
| Low / moderate / high adherence, n | 38 / 54 / 33 | 38 / 54 / 33 | 19 / 47 / 59 | 34 / 53 / 38 |
| MNA × MEDAS categories, $\chi^2(4)$; p | 11.26; 0.024 | 11.26; 0.024 | 8.27; 0.082 † | 10.04; 0.040 |
| Cramér's V | 0.21 | 0.21 | 0.18 | 0.20 |
| Adjusted OR, moderate vs. low (95% CI) | 2.67 (1.12–6.32) | 2.67 (1.12–6.32) | 1.36 (0.47–3.92) | 2.01 (0.84–4.81) |
| Adjusted OR, high vs. low (95% CI) | 4.68 (1.71–12.78) | 4.68 (1.71–12.78) | 3.25 (1.12–9.46) | 4.28 (1.61–11.39) |
| Spearman ρ, MNA vs. MEDAS score (95% CI) | 0.304 (0.13–0.46) | 0.304 (0.13–0.46) | 0.304 (0.13–0.46) | 0.299 (0.13–0.45) |
| Adjusted OR per MEDAS point (95% CI) | 1.40 (1.15–1.72) | 1.40 (1.15–1.72) | 1.40 (1.15–1.72) | 1.50 (1.20–1.89) |
| **Cross-tabulations with the cut-offs <6, 6–7, ≥8, n (low / moderate / high)** | | | | |
| Malnourished (n = 19) | | | 5 / 10 / 4 | 9 / 8 / 2 |
| At risk of malnutrition (n = 62) | | | 10 / 23 / 29 | 18 / 27 / 17 |
| Normal nutritional status (n = 44) | | | 4 / 14 / 26 | 7 / 18 / 19 |

No participant met the wine criterion (item 8), so all participants scored 0 on this item. Excluding it does not change any total score; continuous analyses are therefore unaffected. * Scores multiplied by 14/13 before applying the original cut-offs; for integer scores this yields categories identical to the original ones. † Two expected cell counts were <5 (minimum 2.9). ‡ Item 1 is scored with a frequency criterion in the Turkish adaptation (see Supplementary Table S2). Without items 1 and 8, 12 items varied; the proportionally rescaled cut-offs (7 × 12/14 = 6.0 and 9 × 12/14 = 7.7) are <6, 6–7, and ≥8. Adjusted ORs are from ordinal logistic regression adjusted for age, sex, education, diabetes mellitus, chronic kidney disease, current smoking, and diuretic use.

**Table S7.** Robustness of the ordinal logistic regression estimates.

| Specification | OR (95% CI) / value | *p* |
| --- | --- | --- |
| **A. MEDAS score (per point): alternative specifications** | | |
| Main model (n = 125) * | 1.40 (1.15–1.72) | 0.001 |
| Bootstrap percentile 95% CI (2000 resamples) | 1.18–1.80 | — |
| Unadjusted | 1.40 (1.16–1.70) | 0.001 |
| Adjusted for age and sex only | 1.42 (1.17–1.72) | <0.001 |
| Main model + BMI | 1.40 (1.15–1.72) | 0.001 |
| Main model + LVEF | 1.40 (1.14–1.72) | 0.001 |
| Recorded CKD replaced by eGFR <60 mL/min/1.73 m² (n = 121) | 1.43 (1.16–1.77) | 0.001 |
| Binary logistic regression: normal vs. at risk/malnourished | 1.36 (1.08–1.72) | 0.009 |
| **B. MEDAS categories (reference: low adherence)** | | |
| Moderate vs. low: OR (95% CI); bootstrap 95% CI | 2.67 (1.12–6.32); 1.10–7.91 | 0.026 |
| High vs. low: OR (95% CI); bootstrap 95% CI | 4.68 (1.71–12.78); 1.93–16.36 | 0.003 |
| **C. Effect modification (ratio of ORs for MEDAS per point)** | | |
| MEDAS × female sex | 1.22 (0.80–1.86) | 0.366 |
| MEDAS × diabetes mellitus | 0.96 (0.66–1.41) | 0.842 |
| MEDAS × diuretic use | 0.82 (0.55–1.23) | 0.339 |
| **D. Model diagnostics** | | |
| Outcome categories: malnourished / at risk / normal | 19 / 62 / 44 | |
| Convergence (main models and all bootstrap resamples) | Achieved | |
| Proportional odds assumption (likelihood-ratio test) | — | 0.746 |
| Variance inflation factors (range) | 1.02–1.20 | |
| Largest Spearman \|ϱ\| between predictors (MEDAS vs. current smoking) | 0.29 | |

* Ordinal logistic regression (proportional odds) for a better MNA category, adjusted for age, sex, education (≥high school), diabetes mellitus, chronic kidney disease, current smoking, and diuretic use. All other specifications use the same covariates unless stated otherwise. CKD, chronic kidney disease; eGFR, estimated glomerular filtration rate (2021 CKD-EPI); OR, odds ratio.

**Table S8.** Hemoglobin and anemia according to MNA nutritional-status category (participants with available hemoglobin, $n = 81$).

| | Malnourished | At risk | Normal | *p* |
|---|---|---|---|---|
| n | 11 | 37 | 33 | |
| Hemoglobin (g/dL), mean ± SD | 11.1 ± 1.9 | 11.3 ± 2.1 | 11.8 ± 2.3 | |
| Hemoglobin (g/dL), median (IQR) | 11.6 (10.2–12.6) | 11.3 (9.5–12.9) | 11.7 (9.6–13.3) | 0.671 * |
| Anemia, n (%) † | 9 (81.8) | 26 (70.3) | 21 (63.6) | 0.517 ‡ |
| **Association between hemoglobin and total MNA score** | | | | |
| Spearman ϱ (95% CI) | 0.047 (−0.17 to 0.26) | | | 0.679 |
| β per MNA point, adjusted for age, sex, and eGFR (95% CI), n = 79 | 0.04 (−0.07 to 0.15) | | | 0.503 |
| **Participants with vs. without available hemoglobin** | | | | |
| MEDAS score, median (IQR) | 8 (7–9) vs. 7 (5–8) | | | 0.027 |
| MNA score, median (IQR) | 23.0 (19.0–25.0) vs. 21.8 (18.0–23.6) | | | 0.259 |

* Kruskal–Wallis test. † WHO thresholds: hemoglobin <13 g/dL in men and <12 g/dL in women. ‡ Pearson chi-square test (two expected cell counts <5). Comparisons of participants with and without hemoglobin used the Mann–Whitney U test. These analyses were exploratory.
